# Deploying the Substitutive and Complementary Approaches to Enhance Predictions of Health Behaviour in Health Economics: A Quantitative Study

**DOI:** 10.3390/healthcare13091007

**Published:** 2025-04-27

**Authors:** Damien S. E. Broekharst, Sjaak Bloem, Edward A. G. Groenland, Patrick P. T. Jeurissen, Michel van Agthoven

**Affiliations:** 1 Center for Marketing & Supply Chain Management, Nyenrode Business University, 3621 BG Breukelen, The Netherlands; damienbroekharst@hotmail.com (D.S.E.B.);; 2 IQ Healthcare, Radboud University Medical Center, 6525 GA Nijmegen, The Netherlands; 3 Janssen-Cilag B.V., Johnson & Johnson, 4837 DS Breda, The Netherlands

**Keywords:** expected utility, experienced utility, projected utility, health preference, health experience, health behaviour

## Abstract

**Introduction:** The standard approach to predicting health behaviour in health economics depends on the deployment of expected utility as a predictor. However, this standard approach only explains limited variance in health behaviour, prompting the proposition of the substitutive and complementary approaches. Until now, both approaches have not yet been empirically examined. Hence, this study explores whether the substitutive and complementary approaches enhance predictions of health behaviour compared to the standard approach. **Methods:** Questionnaires were administered online to a panel of Dutch citizens (N = 2550). The questionnaire consisted of items on sample characteristics and instruments on expected utility, experienced utility, projected utility, and health behaviour. Data were analysed using descriptive, reliability, validity, and model statistics. **Results:** The findings of this study suggest that the standard approach explains only limited variance in health behaviour (R^2^ = 0.27). The findings of this study also suggest that the substitutive approach (R^2^ = 0.35–0.42) and complementary approach (R^2^ = 0.38–0.43) explain more variance in health behaviour than the standard approach. The findings of this study further suggest that there are no discernible differences between the substitutive and complementary approaches to predicting health behaviour in health economics. **Conclusions:** Predictive enhancement of the standard approach can be established by applying the substitutive or complementary approach. However, within these approaches, different configurations of predictors might be selected based on different perspectives. Although perspectives may vary, projected utility has a particularly strong effect on and explained variance in health behaviour, which may be especially useful for improving health policies.

## 1. Introduction

Since the inception of health economics as a legitimate field of inquiry, a primary objective of health economists has been to predict health behaviour, enabling them to establish effective interventions while ensuring cost efficiency and the optimal allocation of resources [1,2]. The standard approach to predicting health behaviour in health economics has been defined by the sole deployment of expected utility as a predictor [1,2]. Expected utility in health economics refers to a representation of a weighted preference for a given health-related outcome on a cardinal numerical scale, where a value of 1.0 represents full health, 0.0 represents death, and negative values represent states worse than death [3,4]. Although this standard approach might be considered the most pragmatic, well-established, and broadly recognized approach to predicting health behaviour in health economics, it is not necessarily the most meticulous, accurate, and efficacious, as several studies have shown that the standard approach only explains limited variance in health behaviour [5]. Behavioural economists and psychologists such as Daniel Kahneman and Amos Tversky have argued that this may be due to the ambiguous theoretical axioms underlying the standard approach which are only acceptable under particular circumstances [4,6,7,8]. These authors indicate that the standard approach is based on an assumption of complete information, while research suggests a human inability to assemble and comprehend all available information [4,6,7,8]. Such authors also argue that the standard approach is based on an assumption of absolute rationality, while research shows a human proclivity to decide and act on bounded rationality [4,6,7,8]. They subsequently argue that the standard approach is based on an assumption of stable preferences, while research indicates a human inclination to reconsider health preferences when other choices are offered [4,6,7,8].

In order to account for such limitations, alternative approaches have been proposed that might enhance predictions of health behaviour in health economics, videlicet, the substitutive approach and the complementary approach [9]. The substitutive approach constitutes the replacement of expected utility with one or more other types of utility [9]. The complementary approach constitutes the conjunctive use of expected utility with one or more other types of utility [9]. Types of utility that are most often recommended for substitution or complementarity are experienced utility and projected utility, as they do not suffer the aforementioned theoretical limitations of preference-based methods [9]. Experienced utility refers to the hedonic impact of past health states, while projected utility refers to the realistic approximation of future health states [4,7,10,11,12]. Despite fervent and ardent debates in the scientific literature on this topic, no decisive or conclusive endeavours have been undertaken in order to examine whether these two alternative approaches using experienced utility and projected utility actually enhance predictions of health behaviour in health economics. Hence, this study explores whether the substitutive approach and complementary approach enhance predictions of health behaviour in health economics compared to the standard approach. The findings of this study might legitimize and substantiate the use of one or both alternative approaches in health economics. This may provide health economists, policymakers, and other public health practitioners with an even more solid and robust foundation for performing health economics, albeit after consideration of the pragmatic implications.

## 2. Methods

### 2.1. Research Design

This is a cross-sectional quantitative study employing online questionnaires. The questionnaires were distributed using e-mail among a large panel of Dutch citizens composed by the IPSOS research agency. The panel members were comprehensively informed about the study and were asked for their written consent before participation. Panel members were only included in this study if they were 18 years or older and consented to the use of their responses for research purposes. The questionnaires were distributed among the panel between September and December 2021. The questionnaires consisted of various items on sample characteristics as well as measurement instruments on expected utility, experienced utility, projected utility, and health behaviour.

### 2.2. Data Collection

Items on ‘age’, ‘gender’, ‘health state’, ‘living area’, ‘residential region’, ‘education level’, and ‘annual income’ were used to describe the final sample. These items were measured on nominal scales containing binary response categories as well as ordinal scales containing ascending response categories. These items also served as control variables, except for ‘living area’ and ‘annual income’, as these were similar to other included control variables and it was not desirable to overfit the model. The EuroQol Five-dimensions Five-level (EQ-5D-5L) instrument was used to measure expected utility [13,14,15]. The dimensions (i.e., ‘mobility’, ‘self-care’, ‘usual activities’, ‘pain and discomfort’, ‘anxiety and depression’) were measured with a 5-point scale resulting in generic health state profiles to which standard collective expected utility values were assigned based on a large-scale preference-based time-trade-off study among the Dutch population [16,17]. The original Subjective Health Experience (SHE) ladders were used to measure experienced utility [18,19,20,21]. The dimensions (i.e., ‘physical health’, ‘psychological health’, ‘social health’, ‘general health’) were measured by marking one of 11 levels in which level 0 indicates the worst day of the previous month and level 11 indicates the best day of the previous month [18,19,20,21]. The Subjective Health Experience (SHE) ladders modified for eliciting projections were used to determine projected utility [18,19,20,21]. The dimensions (i.e., ‘physical health’, ‘psychological health’, ‘social health’, ‘general health’) were determined by marking one of 11 levels in which level 0 indicates an approximation of the worst day of next month and level 11 indicates an approximation of the best day of next month [18,19,20,21]. The BRAVO health behaviour dimensions were deployed in order to measure health behaviour [22]. The dimensions (e.g., ‘exercise’, ‘nutrition’, ‘rest’, ‘smoking’, ‘alcohol use’, ‘general health’) were measured on a 6-point Likert scale ranging from 1 = fully disagree to 6 = fully agree [22].

### 2.3. Data Analysis

The items describing the final sample were analysed using descriptive statistics and percentages were reported for the categorical variables, while means were reported for the continuous variables. The control variables that were recoded or originally measured as continuous or dichotomous, namely, ‘age’, ‘gender’, and ‘health state’, were directly included in the models. For categorical variables, dummy variables were created, with ‘education unknown’ as the reference group for ‘education level’ and ‘south’ as the reference group for ‘residential region’. The scale and composite reliability of the instruments were analysed using Cronbach’s alpha (α), rho_a and rho_c coefficients, which were considered reliable if their values surpassed 0.70 [23]. The construct validity of the instruments was analysed using confirmatory factor analysis and their factorial structure was refined by removing items that exhibited double or triple factor loadings (FL) [23]. The convergent validity of the instruments was analysed using the average variance extracted (AVE) coefficient, which was considered sufficient if its value surpassed 0.50 [23]. The discriminant validity of the instruments was analysed using the heterotrait–monotrait (HTMT) ratio, which was considered sufficient if it remained below 0.90 [23]. The effect sizes were analysed using Standardized Beta coefficients (β), which were considered small if below 0.30, average if between 0.30 and 0.50, and large if above 0.50 [23]. The significance levels were analysed using *p*-values, which were considered significant if below 0.05 [23]. The explained variance was analysed using R-squared (R^2^) coefficients, which were considered small if below 0.30, average if between 0.30 and 0.50, and large if above 0.50 [23]. The multicollinearity assumption regarding the independent and dependent variables was checked using the variance inflation factor (VIF) coefficient, which was considered sufficient if its value remained below 5 [23]. The linearity assumption was checked using scatter plots modelling the relationship between the independent and dependent variables [23]. The normality assumption does not need to be satisfied within partial least squares structural equation modelling (PLS-SEM) as it is a nonparametric method capable of handling non-normally distributed variables [23,24]. Software package IBM SPSS Statistics Version 27 was used to describe the final sample and software package SmartPLS Version 4.0 was deployed to analyse the instruments and the models [25,26].

## 3. Results

### 3.1. Sample Characteristics

The final sample analysed in this study was comprised of 2550 panel members. The final sample had a close resemblance to the general population of the Netherlands with regard to the distribution of age, gender, health status, living area, residential region, education level, and annual income [27,28,29,30,31,32,33]. Nevertheless, in the final sample, a slight bias was observed towards a population with higher levels of urbanization compared to the Dutch general population [27,28,29,30,31,32,33]. The sample characteristics are described in Table 1.

### 3.2. Instrument Characteristics

The instruments on experienced utility (α = 0.86; rho_a = 0.86; rho_c = 0.90), projected utility (α = 0.88; rho_a = 0.89; rho_c = 0.92), and health behaviour (α = 0.72; rho_a = 0.79; rho_c = 0.82) are reliable because the reliability coefficients exceeded 0.70. The instrument on experienced utility had sufficient construct validity as all items were loaded on a singular component (FL = 0.79–0.87). The instrument on projected utility had sufficient construct validity as all items were loaded on a singular component (FL = 0.81–0.88). The instrument on health behaviour also had sufficient construct validity as all items were loaded on a singular component (FL = 0.68–0.77) after excluding two items (i.e., ‘alcohol use’, ‘smoking’). The instruments on experienced utility (AVE = 0.70), projected utility (AVE = 0.74), and health behaviour (AVE = 0.54) had sufficient convergent validity because the average variance extracted exceeded 0.50. The instruments on expected utility and experienced utility (HTMT = 0.60), expected utility and projected utility (HTMT = 0.63), expected utility and health behaviour (HTMT = 0.53), experienced utility and projected utility (HTMT = 0.87), experienced utility and health behaviour (HTMT = 0.68), and projected utility and health behaviour (HTMT = 0.73) had sufficient discriminant validity as the heterotrait–monotrait ratios remained below 0.90. As the instrument on expected utility generates unidimensional expected utility values, it was not possible to determine reliability or convergent validity coefficients. Nevertheless, given the aforementioned indicators, the instruments used in this study may be considered valid and reliable.

### 3.3. Model Characteristics

The standard, substitutive, and complementary approaches to predicting health behaviour in health economics are examined and compared using seven models that each deploy expected utility, experienced utility, and projected utility in different configurations. The multicollinearity assumption regarding the independent and dependent variables was satisfied, as the variance inflation factor remained below 5. The linearity assumption was also satisfied as the scatterplots showed a linear relationship between the independent and dependent variables. It should be mentioned that the theoretically assumed relationships in the models were relatively strongly supported by empirical evidence, while this was not the case when these relationships were inverted, decreasing the probability of reverse causality.

#### 3.3.1. Standard Approach

**Figure 1:**
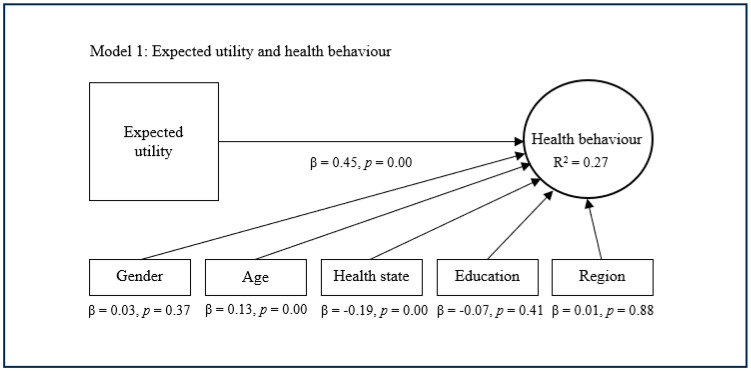
Standard approach with expected utility.

The standard approach is represented by the first model, which deploys expected utility as a main predictor of health behaviour (Figure 1). This first model indicates that expected utility (β = 0.45, *p* = 0.00) has a significant and relatively strong direct effect on health behaviour. This model also indicates that expected utility explains limited variance with regard to health behaviour (R^2^ = 0.27). In this model, the control variables age and health state showed a small significant effect on health behaviour, while the other control variables were nonsignificant.

#### 3.3.2. Substitutive Approach

**Figure 2:**
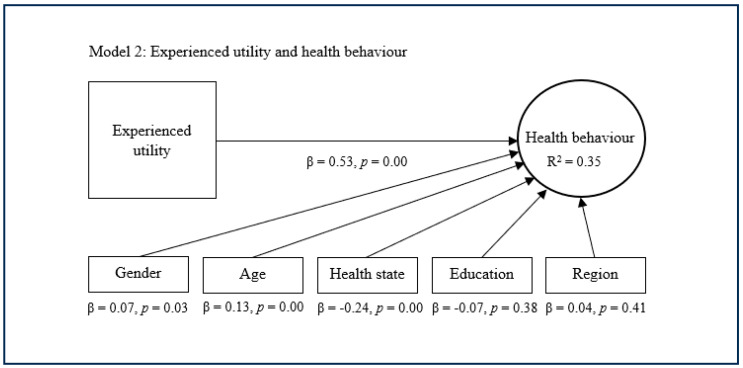
Substitutive approach with experienced utility.

**Figure 3:**
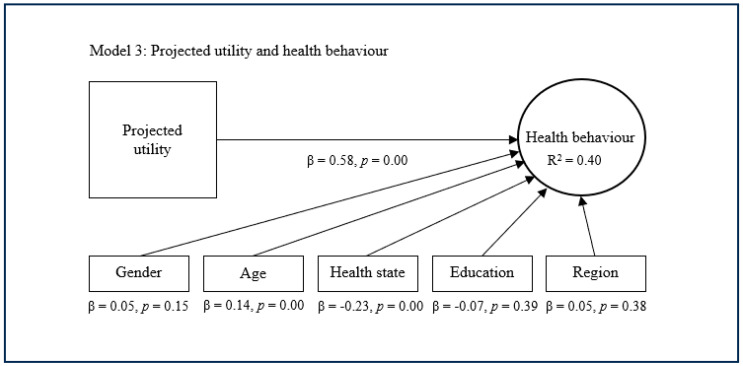
Substitutive approach with projected utility.

**Figure 4:**
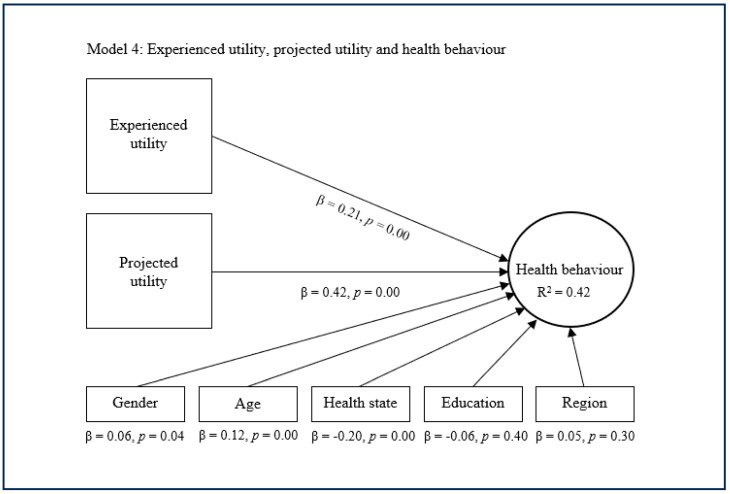
Substitutive approach with experienced and projected utility.

The substitutive approach encompasses the second, third, and fourth models, which replace expected utility with experienced utility and/or projected utility as main predictors of health behaviour (Figure 2, Figure 3 and Figure 4). The second model suggests that experienced utility (β = 0.53, *p* = 0.00) has a significant and quite strong direct effect concerning health behaviour. This model subsequently suggests that experienced utility explains decent variance in health behaviour (R^2^ = 0.35). The third model shows that projected utility (β = 0.58, *p* = 0.00) has a significant and rather strong direct effect regarding health behaviour. This model additionally shows that projected utility explains a decent variance in health behaviour (R^2^ = 0.40). The fourth model indicates that experienced utility (β = 0.21, *p* = 0.00) and projected utility (β = 0.42, *p* = 0.00) combined have a significant and strong direct effect on health behaviour. This model further indicates that experienced utility and projected utility combined explain considerable variance in health behaviour (R^2^ = 0.42). In these models, the control variables age, health state and sometimes gender showed a small significant effect on health behaviour, while the other control variables were nonsignificant.

#### 3.3.3. Complementary Approach

**Figure 5:**
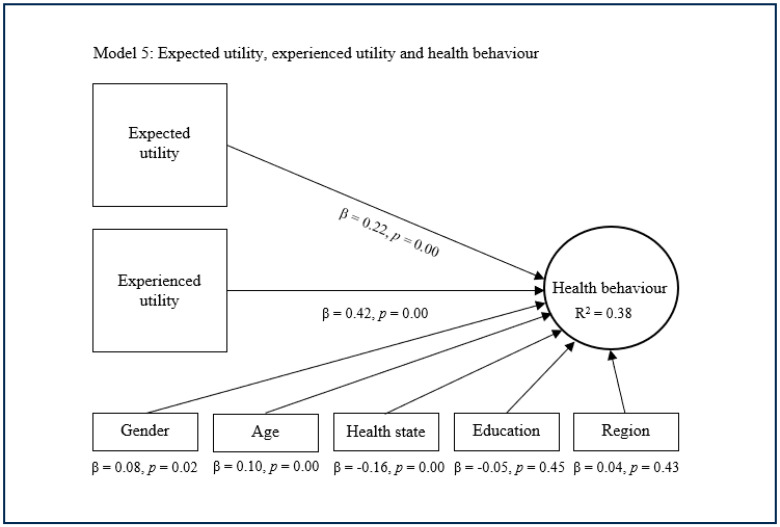
Complementary approach with expected and experienced utility.

**Figure 6:**
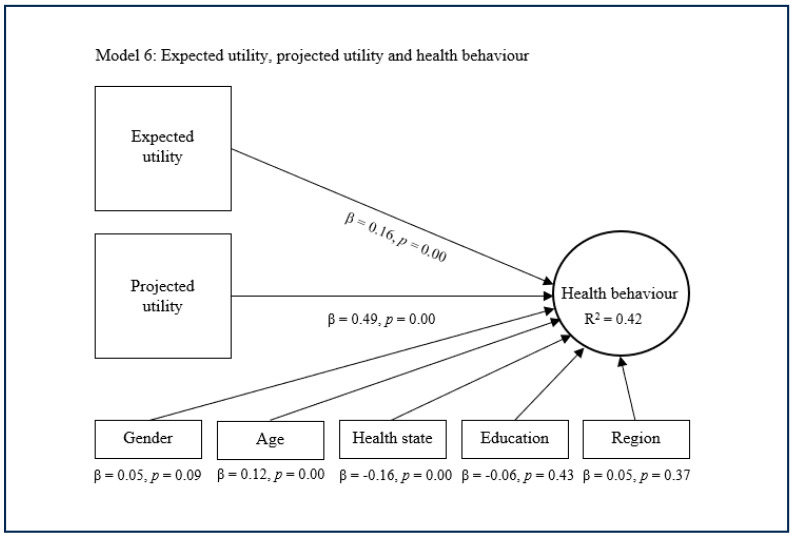
Complementary approach with expected and projected utility.

**Figure 7:**
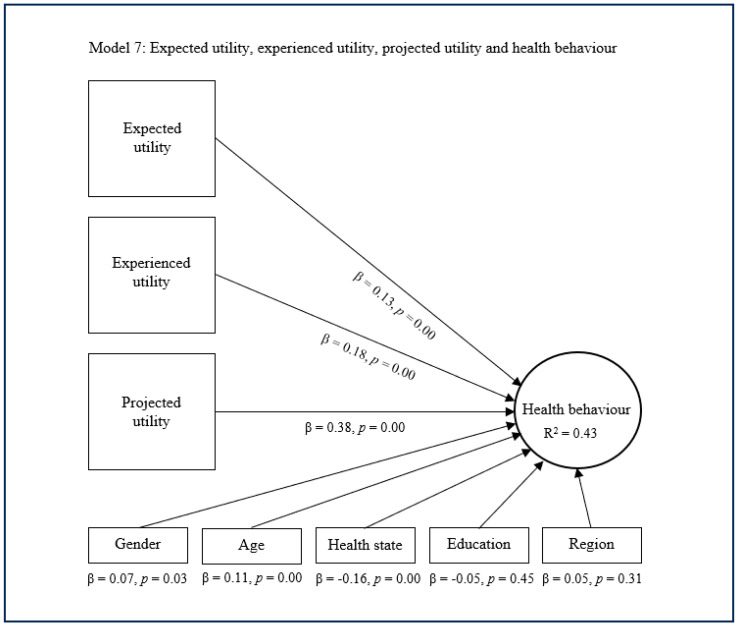
Complementary approach with expected, experienced, and projected utility.

The substitutive approach encompasses the fifth, sixth, and seventh models, which conjunctively deploy expected utility with experienced utility and/or projected utility as main predictors of health behaviour (Figure 5, Figure 6 and Figure 7). The fifth model suggests that expected utility (β = 0.22, *p* = 0.00) and experienced utility (β = 0.42, *p* = 0.00) combined have a significant and quite strong direct effect concerning health behaviour. This model also suggests that expected utility and experienced utility combined explain decent variance in health behaviour (R^2^ = 0.38). The sixth model shows that expected utility (β = 0.16, *p* = 0.00) and projected utility (β = 0.49, *p* = 0.00) combined have a significant and rather strong direct effect regarding health behaviour. This model subsequently shows that expected utility and projected utility combined explain considerable variance in health behaviour (R^2^ = 0.42). The seventh model indicates that expected utility (β = 0.13, *p* = 0.00), experienced utility (β = 0.18, *p* = 0.00), and projected utility (β = 0.38, *p* = 0.00) combined have a significant and strong direct effect on health behaviour. This model additionally indicates that expected utility, experienced utility, and projected utility combined explain considerable variance in health behaviour (R^2^ = 0.43). In these models, the control variables age, health state and sometimes gender showed a small significant effect on health behaviour, while the other control variables were nonsignificant.

## 4. Discussion

This study explored whether the substitutive approach and complementary approach using experienced utility and projected utility enhance predictions of health behaviour in health economics compared to the standard approach. The findings of this study suggest that the standard approach only explains limited variance in health behaviour. The results of this study also suggest that both the substitutive approach and complementary approach have a larger significant direct effect on and explain more variance in health behaviour than the standard approach. The findings of this study further suggest that there are no discernible differences between the substitutive approach and complementary approach to predicting health behaviour in health economics. Given the results of this study, it has become evident that health economists, policymakers and other public health practitioners are well advised to at least consider the substitutive approach and complementary approach to predicting health behaviour in health economics. However, within these substitutive and complementary approaches, the exact configuration of predictors that should be selected for deployment in practice is still up for debate and might differ depending on different applications, priorities, and perspectives. From a theoretical perspective, one might suggest that deploying expected utility as a predictor of health behaviour in any configuration (e.g., models 1, 5, 6, and 7) is unsound and illogical due to the questionable theoretical axioms underlying this concept, such as assumptions of complete information, absolute rationality, and stable preferences [4,6,7,8]. From the same theoretical perspective, one might suggest that deploying expected utility and projected utility together as predictors of health behaviour (e.g., models 6 and 7) is especially unnecessary because both concepts are basically focused on the appraisal of future health states [34]. From a pragmatic perspective, one might suggest that deploying experienced utility and/or projected utility instead of expected utility as predictors of health behaviour (e.g., models 2, 3, and 4) could be confusing and counterproductive because the latter is currently recognized as the global golden standard [16,17]. From the same pragmatic perspective, one might also suggest that deploying both experienced utility and projected utility in conjunction with expected utility as predictors of health behaviour (e.g., model 7) could be cost-, time-, and labour-intensive because many additional measurements have to be conducted and integrated into current health economics [35,36]. From a methodological perspective, one might suggest that deploying expected utility, experienced utility, and projected utility all as predictors of health behaviour (e.g., model 7) without a sufficient increase in explained variance indicates high levels of covariance, violating the principle of parsimony in predictive models [37,38]. In addition to the aforementioned considerations and perspectives (Table 2), it should be remarked that projected utility has a particularly strong effect on and explained variance in health behaviour. This may be explained by the focus of projected utility on eliciting realistic approximations and beliefs about an individual’s future health state, which might inspire actual health behaviour more than hypothetical preferences or even past experiences [10,11,12]. Therefore, the substitutive or complementary deployment of projected utility as a predictor of health behaviour in health economics is likely to be particularly beneficial to health economists, policymakers, and other public health practitioners as it could help them to predict health behaviour more accurately, enabling them to establish and enact more accurate and effective health policies in the future.

### 4.1. Strengths and Limitations

For this study, several strengths and limitations can be identified. The first strength of this study is that it conceptualizes, operationalizes, and measures two alternative approaches to predicting health behaviour in health economics which were until now only hypothesized. The second strength of this study is that it identified and deployed reliable and valid instruments in order to measure the core concepts of this study, which are often deemed difficult to measure. The third strength of this study is that it analyses a sizeable final sample that represents an accurate cross-section of the Dutch population in terms of core demographics. Nonetheless, the final sample of this study might also be a source for some potential limitations as it only focuses on a Dutch population and presents a slight bias towards respondents with slightly higher levels of urbanization, making the results of this study less generalizable. The cross-sectional nature of this research might pose another limitation, as this type of research does not account for possible changes in the measured relationships between model variables over time.

### 4.2. Practical Implications

The results of this study have several implications for health economic practice. If the substitutive approach is applied in practice, it implies that new guidelines for conducting health economic evaluations have to be established and followed. If the complementary approach is applied in practice, it implies that current health economic valuation procedures and studies should take into account the additional measurement and integration of other types of utility. Although these changes in or expansions of measurement and valuation efforts might imply that health economic evaluations become more cost-, time-, and labour-intensive, they could also generate results that are more accurate, precise, and realistic.

### 4.3. Future Research

The results of this study inspire several avenues for further exploration that might be worth pursuing. If a substitutive approach is pursued, an important avenue for further exploration should be addressed that relates to investigating a roadmap to replacing the current type of utility measurement and valuation with a new type of utility measurement and valuation. If a complementary approach is pursued, another important avenue for further exploration should be addressed that relates to investigating ways to integrate different types of utility measurements, values, and dimensions into one composite indicator that can be deployed in health economic theory and practice. If either a substitutive approach or complementary approach is pursued, a final important avenue for further exploration should be addressed that relates to examining and co-creating new guidelines and requirements for conducting health economic evaluations and conceiving of, for instance, quality-adjusted life years.

## 5. Conclusions

During this study, it has become clear that the standard approach to predicting health behaviour in health economics accounts for limited explained variance and leaves room for further enhancement. This necessary enhancement can be established by either applying the substitutive approach or the complementary approach, which both explain more variance in health behaviour. However, within these broad predictive approaches, different configurations of predictors might be selected, based on different applications, priorities, and perspectives. Although these considerations may vary, it should be mentioned that maintaining a certain degree of parsimony in the preferred configuration of predictors is necessary for both pragmatic as well as methodological reasons. Moreover, it should be remarked that projected utility has a particularly strong effect on and explained variance in health behaviour, which may be beneficial to the development of more accurate and effective health policies.

## Figures and Tables

**Table 1:** Sample description.

Variables				
Age	$\bar{x}$ 49.3 years (18–89 years)			
Gender	48.6% male	51.4% female		
Health status	37.2% healthy	27.9% 1 disease	34.9% comorbidities	
Living area	56.8% city	34.3% suburb	8.9% rural	
Residential region	10.1% North Netherlands	21.1% East Netherlands	48.1% West Netherlands	20.7% South Netherlands
Education level	31.5% low	29.3% average	38.9% higher	0.4% unknown
Annual income	35.2% < €36,500	35.8% €36,500–€73,000	14.6% > €73,000	14.4% unknown

**Table 2:** Model assessment.

	Theoretical Perspective	Practical Perspective	Methodological Perspective
Standard approach			
Model 1	X	✓	✓
Substitutive approach			
Model 2	✓	X	✓
Model 3	✓	X	✓
Model 4	✓	X	✓
Complementary approach			
Model 5	X	✓	✓
Model 6	X	✓	✓
Model 7	X	X	X

✓ = Sufficient; X = Insufficient.

## Data Availability

The dataset used during this study is available from the corresponding author upon reasonable request.
